# Programmed cell death pathways in hearing loss: A review of apoptosis, autophagy and programmed necrosis

**DOI:** 10.1111/cpr.12915

**Published:** 2020-10-13

**Authors:** Junhao Wu, Jing Ye, Weili Kong, Shouyue Zhang, Yun Zheng

**Affiliations:** ^1^ Department of Otolaryngology, Head and Neck Surgery, West China Hospital Sichuan University Chengdu China; ^2^ College of Biomedical Engineering Sichuan University Chengdu China; ^3^ School of Life Sciences Tsinghua University Beijing China

**Keywords:** apoptosis, autophagy, hearing loss, programmed necrosis

## Abstract

Programmed cell death (PCD)—apoptosis, autophagy and programmed necrosis—is any pathological form of cell death mediated by intracellular processes. Ototoxic drugs, ageing and noise exposure are some common pathogenic factors of sensorineural hearing loss (SNHL) that can induce the programmed death of auditory hair cells through different pathways, and eventually lead to the loss of hair cells. Furthermore, several mutations in apoptotic genes including *DFNA5*, *DFNA51* and *DFNB74* have been suggested to be responsible for the new functional classes of monogenic hearing loss (HL). Therefore, in this review, we elucidate the role of these three forms of PCD in different types of HL and discuss their guiding significance for HL treatment. We believe that further studies of PCD pathways are necessary to understand the pathogenesis of HL and guide scientists and clinicians to identify new drug targets for HL treatment.

## Graphical abstract

The authors review accumulating evidence pointing out that programmed cell death pathways, including apoptosis, autophagy and necrosis, play key roles in ultimate fates of auditory hair cells, when cells suffer adverse factors. These three forms of PCD may jointly decide occurrence of hearing loss.

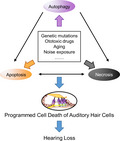

## 1 INTRODUCTION

Hearing loss (HL) is one of the most common sensory defects in humans. The hearing system is complex and depends on the comprehensive functions of many types of tissues and cells in the inner ear. Therefore, mutations in various genes have been proposed to be the cause of HL. It is estimated that 1‐3 of every 1000 newborn children are deaf, and in nearly half of these cases, HL can be attributed to genetic factors.^1^ The well‐known types of acquired HL are ototoxic drug‐induced hearing loss (ODIHL), age‐related hearing loss (ARHL) and noise‐induced hearing loss (NIHL). The pathological characteristics of each type of HL are not the same. The main mechanism of ODIHL is hair cell loss.^2^ The loss of hair, spiral ganglion and vascular striated cells is involved in ARHL.^3^ Noise‐induced hearing loss is caused by excessive exposure to noise.^4^ It involves two main mechanisms, namely mechanical damage and loss of hair cells and spiral ganglia.^4^

Programmed cell death (PCD) appears to play a critical role in the development and diseases of the inner ear. When the nucleus of a cell is affected by severe damage, the initiation of PCD leads to irreversible changes, such as metabolic arrest, structural damage and function loss that can balance cell death and normal cell survival. Several forms of PCD have been found in eukaryotes, including apoptosis, autophagy, programmed necrosis, entosis, ferroptosis, lysosome‐dependent cell death and parthanatos.^5^, ^6^, ^7^, ^8^, ^9^ The contribution of apoptosis in the development of hearing loss has been long studied. Several studies have been conducted to decipher the molecular mechanisms underlying the roles of them in HL. Apoptosis is an ATP‐dependent, enzyme‐mediated, inherently programmed death of cells that are no longer needed or are a threat to the organism.^10^ Apoptosis occurs when DNA molecule in a cell is beyond repair, when a cell receives stress signals from other cells, or when misfolded or unfolded proteins accumulate in a cell. The morphological manifestations of apoptosis include chromatin condensation, cell membrane blebbing, cell shrinkage and apoptotic body formation.^10^ Autophagy is a conserved process of intracellular material turnover in eukaryotes. It is a key mechanism in the response of cells to extracellular or intracellular stress that aid in their survival under certain circumstances; for instance, autophagy protects cells against NIHL by attenuating oxidative stress.^11^ However, overactivation of autophagy may result in cell death.^12^ Specialized double‐membrane vesicles, known as autophagosomes, encapsulate degenerating cytoplasmic organelles or cytosol and subsequently degrade them via the fusion with lysosomes.^13^, ^14^ Necroptosis can be initiated by several factors, and receptor‐interacting proteins (RIPs) 1 and 3 are two key proteins involved in this process. Necroptosis can be morphologically characterized by increased cell volume, swollen organelles, ruptured plasma membrane and the subsequent intracellular content loss.^14^, ^15^ The contents of a ruptured necrotic cell in the interstitial space may trigger inflammation of the adjacent cells.

The pathology of HL has been studied extensively.^16^, ^17^ Recent findings suggest that cellular death mediated via PCD is an important mechanism in HL. Apoptosis and programmed necrosis always lead to cell death, whereas autophagy can lead to cell survival or death. In normal cells, there is a delicate balance between apoptosis‐inducing and apoptosis‐inhibiting factors, and it ensures the survival and proliferation of cells. However, under stress, this balance can be disturbed. The activation of autophagy may play a protective role in the early stages of disease progression. Nevertheless, when the imbalance mediated by pathogenic factors becomes prominent, cells may activate the apoptotic or necrotic death process or over‐activate autophagy leading to cell death. Thus, PCD is important in the development and maintenance of multicellular organisms, and the loss of PCD regulation can lead to diseases. Here, we focused on various causes of HL and the well‐characterized cell death mechanisms (apoptosis, autophagy and necrosis) involved in HL.

## 2 APOPTOTIC PATHWAYS IN HL

**FIGURE 1:**
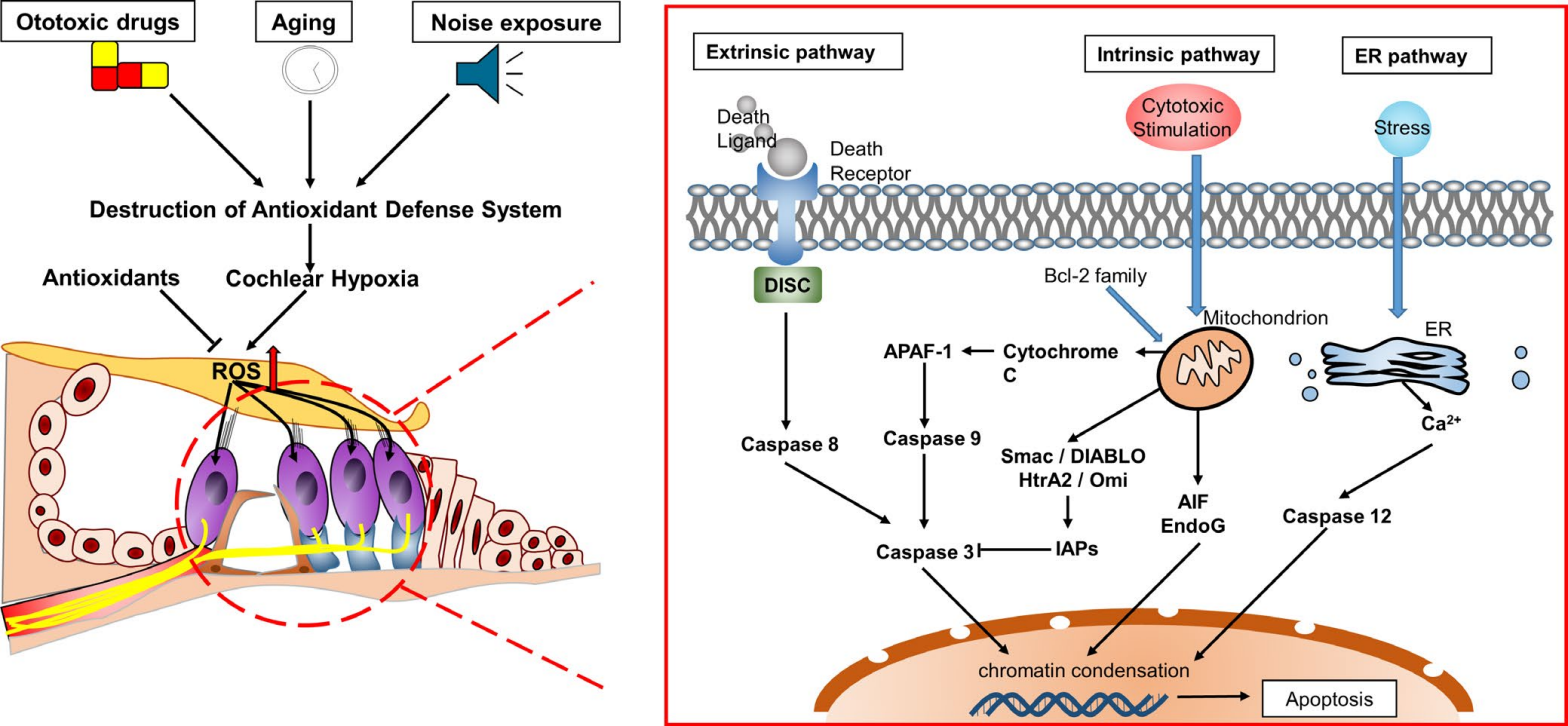
Apoptotic signalling pathways. Factors such as ototoxic drugs, ageing and noise exposure, which lead to hearing loss, damage the antioxidant defence system of the cochlea and cause imbalance of oxidation‐reduction in the inner ear. Reactive oxygen species (ROS) can directly induce the intrinsic apoptosis of cells. Moreover, they can induce the production of cell death ligands to mediate the extrinsic apoptosis process. ROS‐induced intracellular protein damage can cause endoplasmic reticulum stress, which can lead to apoptosis

Apoptosis is an active and highly ordered cell death process regulated by genes (including *Bcl‐2*, *p53* and *c‐Jun*) and a series of enzymes (including caspases and endonuclease G (EndoG)) through intrinsic (mitochondrial), extrinsic (death receptor (DR)) and endoplasmic reticulum (ER) pathways.^18^, ^19^ Apoptosis plays an important role in maintaining the normal growth of an organism. The Bcl‐2 family members are important for the regulation of apoptosis, including Bcl‐2, Bcl‐w, Bax, Bak, Bid and Bad.^20^, ^21^ The initiation of apoptosis depends on the activation of a series of caspases. Caspases can be divided into the following three categories: initiator (caspases initiator 2, 8, 9 and 10), executioner (caspases 3, 6 and 7) and inflammatory (caspases 1, 4 and 5).^22^, ^23^ When a cell is exposed to a fatal stress, apoptosis can be triggered by the initiator caspase 9 or 8 via the mitochondrial or DR pathways. Furthermore, the executioner caspases 3 and 7 are activated, causing the fragmentation of DNA, destruction of nuclear proteins, cytoskeleton and protein cross‐linking, and expression of ligands in phagocytic cells.^24^ In the caspase‐independent pathway, apoptosis‐inducing factor (AIF) and EndoG are released from the mitochondria, and they migrate to the nucleus to condense the chromatin (Figure 1).^22^

ER stress is characterized by the accumulation of misfolded and unfolded proteins, and disruption of calcium and redox balances.^25^ In multicellular eukaryotes, three upstream signalling proteins (IRE1, PERK and ATF6) act as pressure receptors, and they are activated by the level of unfolded proteins in the organelle cavities.^25^ Cells can cope with ER stress by increasing the expression of chaperones and enhancing ER‐associated degradation of misfolded proteins.^26^ However, continued damage can lead to apoptosis (Figure 1). Studies have shown that oxidative stress can induce apoptosis via the DR and mitochondrial pathways.^27^ Reactive oxygen species (ROS) are oxygen free radicals and non‐radical substances, including hydroxyl radicals (OH‐), superoxide anions (O_2_‐), hydrogen peroxide (H_2_O_2_), ozone (O_3_) and singlet oxygen (^1^O_2_) species. Because these ROS contain unpaired electrons, they have a high chemical reactivity. Reactive oxygen species are considered toxic to cell metabolism. An increase in ROS production and the subsequent apoptosis are related to the development of various HL pathologies. These mechanisms suggest that all these factors individually or interactively lead to apoptosis and cochlear damage.

### 2.1 Mutations of apoptosis‐related genes leading to monogenic HL

The different chromosomal loci of nonsyndromic hereditary deafness are designated as deafness (DFN); letters A and B represent autosomal dominant inheritance (*DFNA*) and recessive inheritance (*DFNB*), respectively. Studies on mutant genes responsible for inherited progressive HL have suggested potential mechanisms underlying hair cell apoptosis. Table 1 lists three mutations in apoptotic genes that cause monogenic HL.

**TABLE 1:** Genetic forms of hearing loss (HL)

Molecular function	Gene symbol	Chromosomal locus	Locus name
Nucleus	GSDME	7p15	DFNA5
Tight junctions	TJP2	9q21.11	DFNA51
Mitochondria	MSRB3	12q14.2‐15	DFNB74

Note

DFNA5 is targeted at chromosome 7p15 as the fifth DFNA site that leads to progressive HL, which starts at high frequencies. DFNA51 is caused by a tandem inverted genomic duplication of 270 kb at chromosome 9q21.11. DFNB74 is a novel locus on chromosome 12q14.2‐15 that is responsible for autosomal recessive nonsyndromic hearing impairment.

#### 2.1.1 DFNA5

**FIGURE 2:**
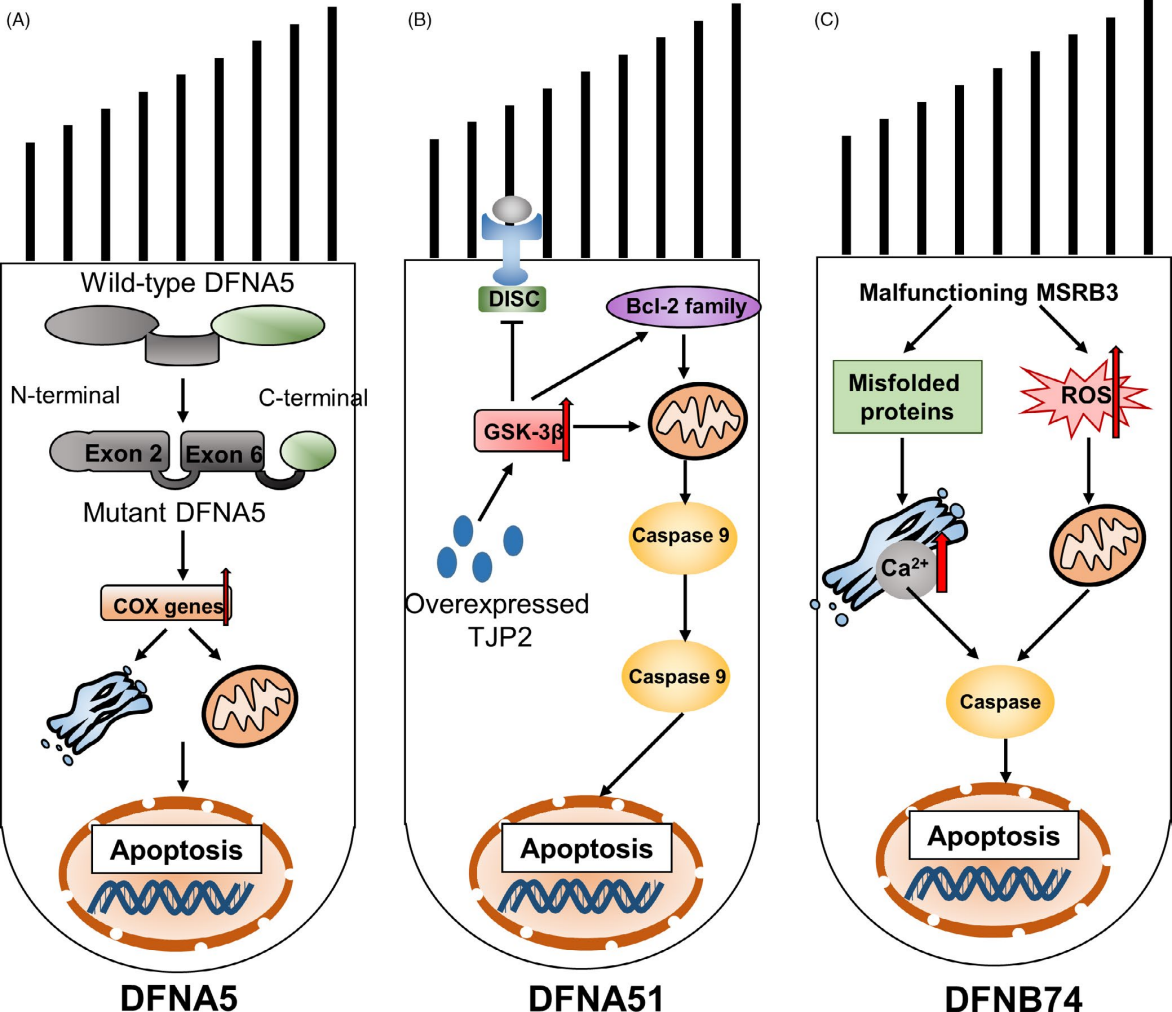
Schematics of three mutations that lead to monogenic hearing loss. A, DFNA5: the apoptosis‐inducing region of DFNA5 is located in exons 2 and 6 of the N‐terminal domain. Skipping exon 8 can change and shorten the C‐terminal domain of DFNA5, reveal the apoptosis‐inducing region and lead to apoptosis. B, DFNA51: overexpression of TJP2 induces apoptosis by activating glycogen synthase kinase 3β (GSK‐3β). C, DFNB74: malfunctioning MSRB3 leads to the accumulation of oxidative damage proteins and reactive oxygen species (ROS), ultimately leading to endoplasmic reticulum stress and subsequent activation of endogenous apoptotic pathways

*DFNA5* is one of the mutated genes related to PCD that leads to sensorineural HL. So far, only intronic mutations have been reported to cause exon 8 skipping in patients with *DFNA5*‐related HL.^28^, ^29^, ^30^, ^31^ The protein encoded by *DFNA5* belongs to the gasdermin superfamily as it contains a gasdermin domain. A previous study reported that wild‐type *DFNA5* (*wtDFNA5*) had no effect on yeast cells, whereas mutant *DFNA5* (*mutDFNA5*) led to cell cycle arrest.^32^ In mammalian cells, the transfection of *mutDFNA5* led mutDFNA5 to cell death, whereas the transfection of *wtDFNA5*wtDFNA5 could not.^33^ Thus, HL caused by *mutDFNA5* can be attributed to functional mutations. In a mutant *DFNA5* cell line, the upregulation of different cytochrome c oxidase (*COX*) genes was found to be associated with cell death mechanisms under oxidative stress.^34^ In the same research model, the downregulation of protein sorting‐ and folding‐related mechanisms indicated that ER stress has a potential role in cell death induced by *DFNA5* (Figure 2A).^34^

#### 2.1.2 DFNA51

*DFNA51* is an inverted genomic duplication of 270‐kb DNA, including the entire wild‐type *TJP2* that encodes the tight junction protein (ZO‐2). ZO‐2 belongs to the membrane‐associated family of guanylate kinase homologs, and it contains 3 PDZ domains, 1 SH3 domain and 1 GUK domain.^35^ ZO‐2 binds to the C‐terminal of the connective transmembrane protein and then connects to the actin in the cytoskeleton and regulates the location of different subtypes of cells by interacting with the signal transduction pathway molecules.^36^ *TJP2* is mainly expressed between the hair and supporting cells in the organ of Corti, and helps maintain the barrier between ductus perilymphaticus and ductus endolymphaticus. Its expression decreases with age. The pathogenic mutation gene is an inverted genomic duplication of *TJP2* that results in the overexpression of TJP2, which leads to autosomal dominant nonsyndromic HL. The expression of *TJP2* mRNA in patients with duplicate genes was approximately 1.7‐fold higher than that in normal controls. The overexpression of *TJP2* in vitro leads to a decrease in the phosphorylation and activation of glycogen synthase kinase 3β (GSK‐3β). GSK‐3β promotes cell death via the mitochondrial intrinsic apoptotic pathway, but it inhibits the DR‐mediated extrinsic apoptotic pathway.^37^ The results of real‐time fluorescent quantitative polymerase chain reaction showed that even a slight increase in the expression of *Bcl‐w* altered the expression of other Bcl‐2 family members and the 18‐kDa translocator protein (TSPO) may shift the overall steady‐state balance towards apoptosis and thus result in HL.^38^ However, the complete loss of *TJP2* can lead to embryonic death; thus, *TJP2* knockout was found to be lethal in mice (Figure 2B).^39^

#### 2.1.3 DFNB74

The mutations c.265 T > G and c.55 T > C in methionine sulfoxide reductase B3 (*MSRB3*) are related to autosomal recessive HL. The mutated gene is also known *DFNB74*. The gene has four isoforms. Isotype A is located in the ER, and the other three isotypes (B, C and D) are located in the mitochondria. *MSRB3* encodes a methionine sulfoxide reductase that is involved in the repair of oxidative damage proteins. In the organ of Corti in mice, the expression of *MSRB3* is upregulated in the inner and outer hair cells, but it is downregulated in the supporting cells. *MSRB3* mutations lead to the disruption of protein functions. This in turn leads to the accumulation of oxidative damage‐related proteins and ROS, activation of caspase and initiation of apoptosis or programmed necrosis. MSRB3 deficiency can also increase the level of cytosolic calcium. The disruption of calcium homeostasis can trigger ER stress and activate Bcl‐2‐like protein 11 (also known as BIM) molecules that promote apoptosis, and thus lead to HL (Figure 2C).^40^, ^41^

### 2.2 Ototoxic drug‐induced hearing loss (ODIHL)

#### 2.2.1 Aminoglycoside antibiotics

Aminoglycosides are broad‐spectrum antibiotics, but their potential ototoxicity needs to be closely monitored.^42^ The ototoxicity of aminoglycosides is irreversible because the hair cells of the cochlea cannot proliferate and recover. Aminoglycosides may damage the hair cells of the cochlea and type I sensory cells of the vestibule by triggering different apoptotic signals, thus resulting in HL and vertigo.^43^ Mitochondrial pathways play a key role in aminoglycoside‐induced apoptosis and may be the main target of these drugs. Aminoglycosides tend to accumulate in the mitochondria of hair cells.^44^ Gentamicin directly inhibits protein synthesis in mitochondrial ribosomes and triggers the opening of mPTP that leads to the release of apoptotic factors.^44^, ^45^ In addition, L‐carnitine promotes mitochondrial function, which can prevent the damage of the outer hair cells after the administration of gentamicin.^46^

ROS has been identified as the main cause of HL mediated by aminoglycosides.^47^ ROS induces mitochondrial damage, thereby leading to the activation of various pathways that lead to apoptosis. Aminoglycosides can accelerate the nonenzymatic formation of ROS by redox active iron complex, and induce the intracellular enzymatic reaction.^48^ Additionally, a previous study demonstrated that dexamethasone, melatonin (MLT) and tacrolimus decrease the levels of ROS in GM‐exposed explants.^49^ Interestingly, the c‐Jun‐NH‐terminal kinase (JNK) cascade reaction combines oxidative stress with apoptosis.^50^ Through in vivo experiments, it has been shown that administration of an aminoglycoside leads to the activation of the JNK pathway, which triggers the apoptosis of cochlear cells. Accordingly, JNK cascade inhibitors, such as CEP‐1347 and estradiol, can reduce the loss of hair cells after the administration of gentamicin.^51^, ^52^ However, Kalinec et al reported that gentamicin ototoxicity is mediated by the inhibition of the JNK pathway.^46^ Evidently, there is no consensus on whether the signalling enzyme is activated by gentamicin ototoxicity.

Hair cells are not the only drug targets. Aminoglycosides also have effects on stria vascularis, including thinning of the tissue and reduction of marginal cells.^53^, ^54^ The degeneration of spiral ganglion cells after aminoglycoside treatment may be attributed to the loss of hair cells innervated by the ganglion cells.^55^ However, some studies have shown that the spiral ganglion can be affected without obvious damage to the hair cells.^56^, ^57^ This suggests the complexity of the damage pattern of aminoglycoside antibiotics.

#### 2.2.2 Cisplatin

The ototoxicity of cisplatin is well known.^58^ The ototoxic effects of cisplatin can be divided into two categories. The first is the reversible inhibition of conduction current, voltage‐dependent calcium current in hair cells and the current response in stria vascularis.^59^ Another persistent toxic reaction induces irreversible changes in cochlear morphology, thus resulting in irreversible, bilateral, high‐frequency HL.^60^ Compared with the inner hair cells, the outer hair cells,^61^ vascular marginal cells and spiral ganglion cells are more easily degenerated.^62^, ^63^ Cisplatin ototoxicity occurs through the formation of ROS in the cochlear tissue, accompanied by changes in potassium conductivity, which lead to cell death.^64^, ^65^ The cochlea has an effective antioxidant defence system. This system includes antioxidants, such as vitamin C, vitamin E and glutathione (GSH), as well as several antioxidant enzymes that are expressed in the cochlea, such as superoxide dismutase (SOD), GSH peroxidase and catalase. Cochleas extracted from cisplatin‐treated animals demonstrated consumption of GSH, reduction of antioxidant enzyme activity and an increase in lipid peroxidation.^66^, ^67^

Cisplatin increased the production of ROS in the inner ear.^68^ It seems that one of the important sources of these ROS is nicotinamide adenine dinucleotide phosphate (NADPH) oxidase 3, which is a type of superoxide that produces NADPH oxidase. It is highly expressed in the Corti organ,^69^ and its level increases after cisplatin treatment.^69^, ^70^ Other NADPH oxidases are also important in the production of ROS in response to cisplatin ototoxicity.^71^ Excessive ROS production may damage the antioxidant defence capacity of cochlear cells. p53 is activated in response to oxidative stress to regulate the expression of genes (eg Bax) that control DNA repair and cell death.^72^ Bax can interact with voltage‐dependent ion channels in mitochondria, which mediate the release of cytochrome c and have the effect of apoptosis.^70^, ^73^ The corresponding targeted drugs have been proved to be suitable for the protection of cisplatin ototoxicity. For example, antioxidant administration in the early stage of cisplatin‐mediated ototoxicity can prevent ROS from having an additional downstream role in the cell death cascade reaction and in the function of reagents. Many of these antioxidants are mercaptan compounds with high‐affinity for platinum, such as N‐acetylcysteine (NAC),^74^ sodium thiosulfate^75^ and D‐methionine.^75^, ^76^ Other antioxidants that are resistant to cisplatin include ebselen, lipoic acid, diethyldithiocarbamate and 4‐methylthiobenzoic acid.^77^ The application of the p53 inhibitor pifithrin‐α in cisplatin‐exposed cochlear organotypic cultures decreased hair cell injury. This was related to the decreased expression of p53 and caspase 3.^78^ Specific caspase 9 and caspase 3 inhibitors can protect auditory hair cells from cisplatin‐induced apoptosis and HL.^79^

Other potential apoptotic pathways in the stria vascularis lateralis or spiral ganglia include increased Bax levels, decreased bcl‐2 expression,^61^ activation of NF‐κB,^80^, ^81^ formation of inducible nitric oxide synthase,^62^, ^82^ activation of the high‐mobility group 1^83^ and production of 4‐hydroxynonenal (4‐HNE).^84^

### 2.3 Age‐related Hearing Loss (ARHL)

The prevalence of ARHL is expected to rise with the increase in the ageing population.^85^, ^86^, ^87^ Although many factors have been studied, including environmental, genetic and medical factors,^88^, ^89^ the precise mechanism of ARHL is unclear. At present, it is generally believed that ARHL is the result of a combination of genetic predispositions and various insults to the inner ear that accumulate during daily activities. ARHL is not the first mock examination in histopathology and pathophysiology. It may be accompanied by the degeneration and loss of sensory hair cells, spiral ganglion cells, stria vascularis cells and basement membrane with age.^90^ Apoptosis in ARHL is mediated via exogenous and endogenous pathways. Exogenous pathways are triggered by ligands that bind to cell surface receptors and may be related to environmental and medical factors.^91^, ^92^ The endogenous pathway is mitochondrial‐dependent and is triggered by the loss of the mitochondrial membrane potential. The prevention of ARHL after the deletion of mitochondrial apoptotic gene (eg Bcl‐2 family member Bak) indicates that the endogenous apoptotic pathway is necessary for progression of ARHL.^93^ It has been demonstrated that in ARHL models of mice, rats and gerbils, apoptosis occurs through the caspase‐dependent pathway, and involves the Bcl‐2 family proteins.^94^, ^95^, ^96^ Immunohistochemical analysis of the cochlea of ageing CBA/J mice showed an increase in the phosphorylation (ie activation) of JNK and p38 MAPK in outer hair cells.^94^ In addition, the same study also proved the release of cytochrome c, activation of caspase 9 and translocation of Endo G in the hair cells of ageing mouse.

ROS play an important role in ARHL.^97^ They may cause DNA damage that leads to the upregulation of p53, which, in turn, leads to the chronic activation of the mitochondrial Bak pathway, finally resulting in apoptosis.^98^ Consistent with this process, in all cell types of Corti organs, levels of antioxidant defence factors such as mitochondrial SOD 2 (SOD2) have been observed to decrease significantly with age, thus indicating that oxidative imbalance leads to ARHL.^99^ Therefore, the role of antioxidant supplementation in ARHL has been studied. In Fischer 344 rats, vitamin C, vitamin E, MLT and lazaroid treatment yielded better results in the maintenance of auditory sensitivity and reduction of the number of mitochondrial DNA (mtDNA) deletions compared to those observed with the placebo.^100^ In addition, C57BL/6J mice provided an antioxidant diet (α‐lipoic acid, coenzyme Q10, NAC) had a much lower ABR hearing threshold than that of control mice.^93^ Nevertheless, Sha et al kept CBA/J mice on a long‐term diet from 10 to 22 months of age and claimed that foods rich in vitamins A, C, E and alpha lipoic acid did not delay or reduce ARHL.^94^ Accordingly, Keithley et al found that transgenic mice expressing SOD2 yielded outcomes that were contrary to expectations, while the HL in mice who were 20 months old was more prominent than that in the parent strain of B6 mice.^101^ These results suggest that mitochondrial ROS may be a factor in ARHL, but there are other factors involved as well.

Mitochondria are particularly susceptible to the accumulation of genetic or environmental damages because—unlike the nucleus—mtDNA is regularly replicating independent of cell cycle replication and lacks an effective DNA repair system and protective histones. Therefore, the total number of its DNA mutations is higher than that of the accumulation of mitochondrial DNA mutations are thought to cause age‐related degenerative diseases,^102^ and an increase in mitochondrial DNA mutations in human cochlear tissue has also been observed.^103^ The same mechanism was proposed in the mouse ARHL model.^104^, ^105^ The main mutation of mitochondrial DNA occurs in the gene encoding the mitochondrial oxygen phosphorus complex and leads to abnormal oxygen phosphorus activity and mitochondrial dysfunction, and increases in intracellular free calcium. Calpain and cathepsin are released from lysosomes in response to the increased intracellular calcium. They are calcium‐dependent proteases that activate downstream pathways through the proteolysis of target proteins. They are part of cell death signals that are independent of cystatin and are involved in apoptosis and necrotic cell death.^94^ However, the exact pathway of apoptotic activation caused by ARHL has not been clearly defined. In fact, it is possible to activate multiple pathways at the same time because ARHL is the product of a multifactorial process.

### 2.4 Noise‐induced HL (NIHL)

Noise is also the main cause of HL.^106^ After noise exposure, there are two main mechanisms that cause cochlear damage. The first is direct mechanical damage that leads to the loss of hair cells through the mechanical destruction of cilia, and to the damage of supporting and sensory cells.^107^ Another mechanism involves the biochemical pathway that leads to cell death through apoptosis or necrosis. It has been shown that apoptosis is the key mediator of noise‐induced HL. After noise exposure, an increase in chromatin condensation and levels of apoptosis markers^108^, ^109^, ^110^, ^111^, ^112^ such as caspase 3, 8 or 9,^113^ tumour necrosis factor receptor,^111^ and Bcl‐2‐associated death promoters (Bad)^114^ was observed in cochlear cells of guinea pigs, chinchillas and rats models. Wang et al showed that the application of riluzole (inhibitor of apoptosis and necrosis) in the cochlea can protect the cochlea from hearing impairment.^115^, ^116^ These results indicate the importance of the apoptotic pathway in NIHL.

Several ways of inducing apoptosis in NIHL have been studied in animal models. After noise exposure in a guinea pig model, AIF and EndoG entered the cytoplasm of the cochlear cell^117^ and were then transferred to the nucleus, triggering apoptosis in a caspase‐independent manner. Noise exposure triggers the activation of caspase 8 and caspase 9 by exogenous and endogenous pathways, and both apoptosis markers are associated with signalling pathways that lead to caspase 3 activation.^113^ A variety of agents can attenuate NIHL, including iron chelators, antioxidants and vasoactive factors.^118^, ^119^ However, these factors individually have limited protective efficacy. These observations are consistent with the suggestion that noise may induce cell death via both caspase‐dependent and caspase‐independent apoptosis.^117^

After noise exposure ends, ROS or other similar reactive species levels are generally increased.^120^ ROS were observed to be present in the cochlea for an extended period of time following noise exposure.^121^ These species were responsible for the morphological observations of delayed and sustained damages.^121^ Consistent with the hypothesis of ROS formation, antioxidant molecules can be used for protection, such as water‐soluble coenzyme Q10,^122^ NAC,^119^, ^123^ D‐methionine^124^ and GSH,^125^ which will decrease the amount of apoptosis in hair cells after noise exposure. ROS formation can activate the JNK signalling pathway.^126^ In the noise‐damaged guinea pig model, JNK mediates apoptosis,^110^ and blocking of the JNK pathway has a protective effect on noise. Mice exposed to noise contained fewer apoptotic cells than those in the control group, when they were fed with JNK inhibitors such as all transretinoic acid and CEP‐1347 (small molecules derived from indole‐carbazole K252a).^127^, ^128^, ^129^ Another study reported that blocking the JNK pathway with locally delivered D‐JNK‐1 through the round window membrane can prevent hair cell death and permanent NIHL.^110^

In addition, the increase of free Ca^2+^ in the outer hair cells, or the activation of Ca^2+^ and calmodulin‐controlled calcineurin may trigger the apoptosis or necrosis pathway without ROS.^130^, ^131^ Calcium metabolic disorders and free radicals can also cause ER stress. Severe ER stress is more likely to induce the expression of CHOP^132^ and would lead to ER stress‐related apoptosis based on the downregulation of the expression of antiapoptotic proteins such as Bcl‐xL.^133^ Glucocorticoid‐induced leucine zipper protects the cochlea from ER stress‐induced apoptosis following noise exposure by reducing chop and regulating ER stress‐related apoptosis proteins.^134^

## 3 AUTOPHAGIC PATHWAYS IN HL

**FIGURE 3:**
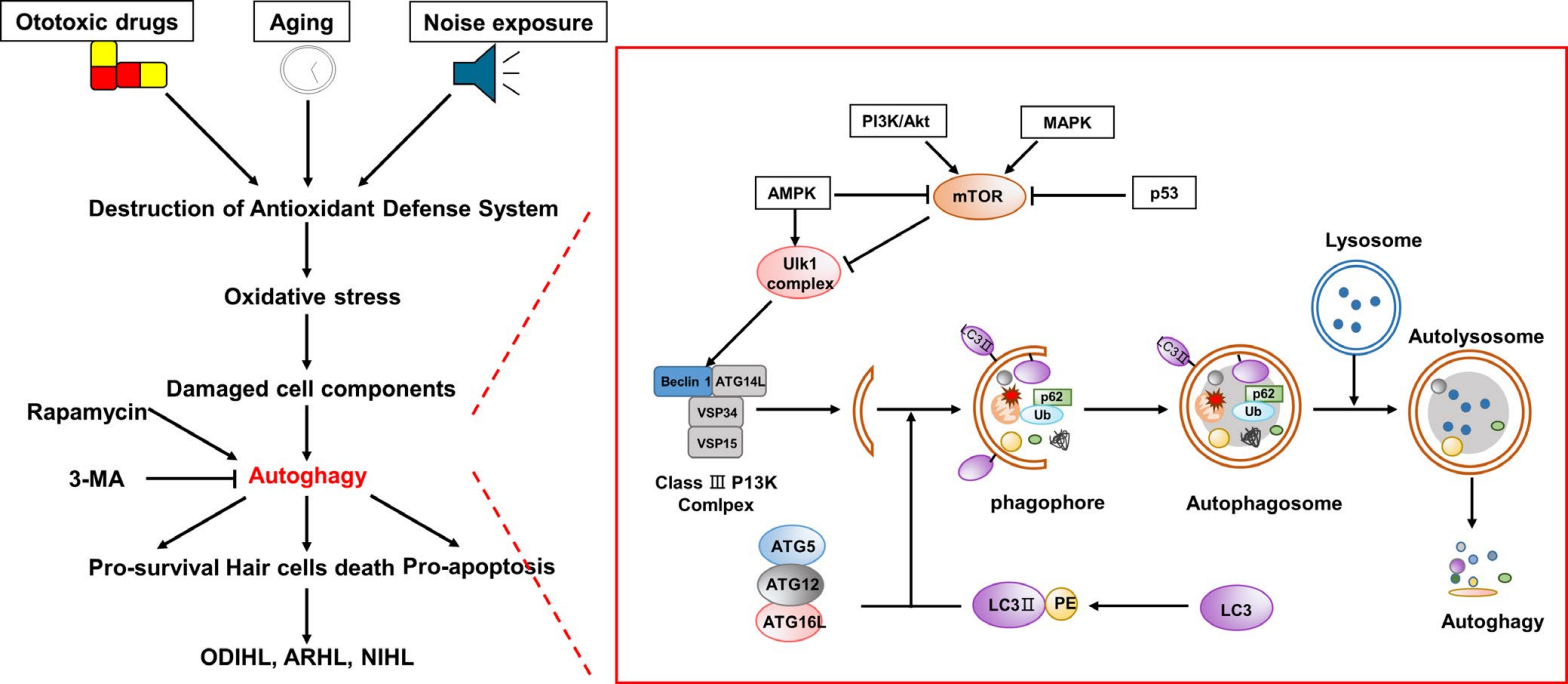
Signalling pathway of autophagy in HL. Unc‐51‐like autophagy activating kinase 1 (ULK1) is phosphorylated upon the activation of AMPK and inactivation of mTOR. The activated ULK1 complex and class III phosphoinositide 3 kinase (PI3K) complex form phosphors. Microtubule‐associated protein 1 light‐chain 3 (LC3) protein can be coupled with phosphatidylethanolamine to form LC3Ⅱ. The complex of ATG5, ATG12, ATG16L and LC3Ⅱ can stimulate the elongation of phagocytes, which provide a platform for the formation of phagosomes. On approaching the ubiquitin protein binding to p60 and LC3Ⅱ, the phagosome closes to form autophagosomes. Further, autophagosomes fuse with lysosomes to form autolysosomes in which the contents are degraded

Autophagy is a protective mechanism triggered to limit pathological changes. In all eukaryotic cells, autophagy processes unnecessary or dysfunctional cell components such as damaged organelles and misfolded proteins^135^ through highly regulated processes.^136^, ^137^ Autophagy is a process that mediates the formation of a bimembrane autophagic body that surrounds the damaged organelle or other cytoplasmic components.^138^ The core molecule of autophagy regulation is the rapamycin kinase mammalian target (mTOR). The pathways activating mTOR such as the Akt and MAPK signalling pathways inhibit autophagy, while the pathways negatively regulating mTOR such as the adenosine 5’‐monophosphate activated protein kinase (AMPK) and p53 signalling pathways promote autophagy.^139^ Under adverse conditions, AMPK is activated and mTOR is inactivated to enter the autophagy pathway. Multiple autophagy‐related (ATG) proteins are involved in this process (Figure 3).

Autophagy is a common cell response to starvation or other stresses. Basic autophagy is important in controlling the cytoplasmic composition and homeostasis of various mitotic cells.^140^ Autophagy is involved in the development of normal cochlea. Based on real‐time PCR, Rodríguez et al found that the peak timepoints of ATG4b, ATG5, ATG9a and Beclin1 in the mouse cochlea were the same as that at which the cochlear was fully functional.^141^ Basic autophagy plays an important role in the maintenance of hair cell morphology and hearing ability. Chisato et al found that ATG5 knockout in mice resulted in the degeneration of auditory hair cells and severe congenital HL. In the hair cells of autophagy‐deficient mice, accumulation of polyubiquitin and p62/SQSTM1 (autophagy matrix) as inclusion bodies was observed at the first week of life.^140^ Therefore, impaired autophagy function can have adverse effects on auditory hair cells. Tsuchihashi et al used low‐dose H_2_O_2_ to construct an auditory cell model; increased phosphorylation of 4EBP1 following H_2_O_2_ treatment led to impaired autophagy function, which in turn resulted in oxidative stress‐induced premature ageing.^142^

Some studies showed that autophagy plays a role in the prevention of hearing impairment, such as NIHL and ODIHL.^11^, ^143^ Previous studies have shown that ROS has the ability to induce autophagy in auditory cells.^11^, ^144^ Yuan et al reported that autophagy reduced NIHL by reducing oxidative stress.^11^ Rapamycin can increase autophagy activity, inhibit ROS accumulation and prevent cell death induced by H_2_O_2_. It significantly increased the expression of microtubule‐associated protein 1 light‐chain 3Ⅱ (LC3Ⅱ), decreased the levels of 4‐HNE and 3‐nitrotyrosine (3‐NT) and reduced NIHL and loss of hair cells. In contrast, LC3B reduction by the autophagy inhibitor 3‐methyladenine (3‐MA) or LC3Ⅱ small interfering RNA increased the levels of 3‐NT in outer hair cells and promoted hair cell loss and NIHL. He et al found that autophagy activity was significantly increased, including enhanced autophagosome‐lysosome fusion, in both cochlear hair cells and HEI‐OC‐1 cells after neomycin or gentamicin injury, suggesting that autophagy might be correlated with aminoglycoside‐induced cell death.^144^ Rapamycin treatment reduced ROS levels and hair cells death induced by aminoglycosides (including cisplatin, gentamicin and neomycin),^144^, ^145^, ^146^ while 3‐MA treatment or ATG5 deletion increased ROS levels and apoptosis.^144^ Chika et al demonstrated that oral administration of low‐dose rapamycin induced autophagy activation in cochlear outer sulcus cells, suggesting that rapamycin could be a feasible drug to manipulate inner ear cells.^147^ Phosphatase and tensin homolog‐induced putative kinase 1 also protected hair cells from cisplatin‐induced ototoxicity following induction of autophagy.^148^

Yin et al found that autophagic activation of HEI‐OC1 cells increased the expression of the nuclear binding domain and leucine‐rich repeat containing family member X1 (NLRX1) in cisplatin‐induced injury.^12^ NLRX1 overexpression led to the amount of accumulation of autophagosomes in HEI‐OC1 cells in normal condition and a higher activation of autophagy concurrent with cell injury in HEI‐OC1 cells treated with cisplatin. These findings suggest that decrease the activation level of autophagy concurrent with increased cell viability in HEI‐OC1 cells treated with cisplatin, while overactivation of autophagy can lead to pathological changes in response to cisplatin exposure.^12^ MicroRNA‐96 is the first microRNA mutation that has been reported to be related to human deafness.^149^ The decreased expression of microRNA‐96 may directly upregulate the expression of ATG7. Excessive activation of autophagy induces degeneration and death of neurons.^150^

## 4 THE PROGRAMMED NECROSIS PATHWAYS IN HL

**FIGURE 4:**
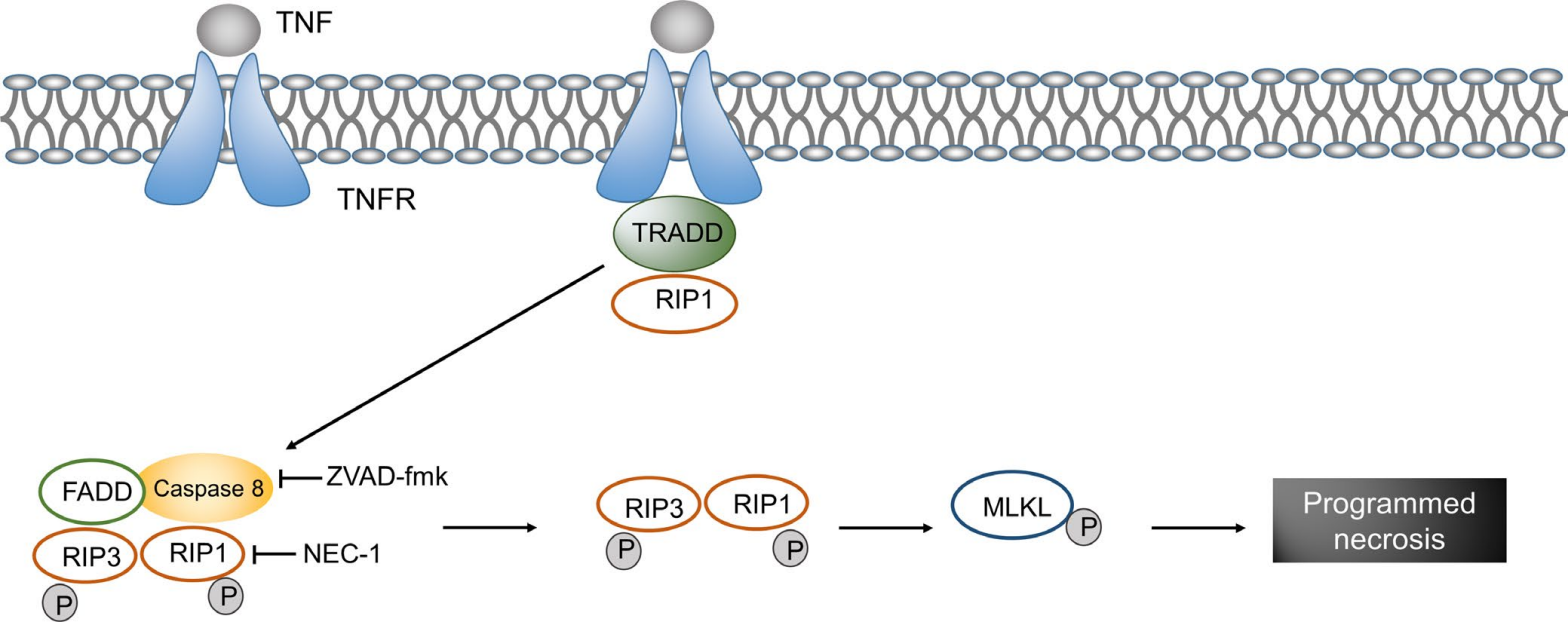
Signalling pathway of programmed necrosis in hearing loss. When a ligand binds to tumour necrosis factor receptor (TNFR), a combination of TNFR‐associated death domain (TRADD) and receptor‐interacting protein (RIP) 1 increases the level of RIP3 and induces self‐ and transphosphorylation, which is followed by the oligomerization of phosphorylated RIP3. Active RIP3 catalyses the phosphorylation of the mixed lineage kinase domain‐like protein (MLKL), thus resulting in the formation of MLKL oligomers and in translocation to the plasma membrane. Through the reversal mechanism, specific phosphatidylinositol phosphates are combined that lead to plasma membrane permeability and eventually cell necrosis

Programmed necrosis is a type of regulatory cell death caused by microenvironmental disorders inside and outside the cells and is detected by specific DRs. The DR is usually a type‐1 tumour necrosis factor receptor,^151^ besides suicide‐related factors (eg Fas) and pathogen recognition receptors (eg toll‐like receptors 3), that can also mediate programmed cell necrosis.^152^, ^153^ RIP1 and RIP3 can mediate the activation of programmed necrosis pathway through physical and functional interactions^154^, ^155^, ^156^, ^157^ (Figure 4).

Zheng et al reported that the pan caspase inhibitor ZVAD blocked noise‐induced caspase 8 activation and reduced apoptosis in outer hair cells but stimulated the accumulation of RIP1 and RIP3 levels, resulting in the depletion of adenosine triphosphate and necrosis of cells.^158^ These findings suggest that a balance between apoptosis and necrosis is required for noise‐induced death of outer hair cells, which is regulated by caspase 8 and RIP kinase. Choi et al found that treatment with Nec‐1, a selective RIP1 inhibitor, significantly inhibited cisplatin‐induced cell death in HEI‐OC1 cells, while the use of ZVAD did not change cisplatin‐induced cell death in HEI‐OC1 cells.^159^ Their results suggested that RIP3‐dependent cell necrosis may mediate cisplatin ototoxicity.^159^ Douglas et al studied the ototoxicity of aminoglycosides and cisplatin in a murine model^160^ and suggested that the main form of hair cell death induced by aminoglycosides and cisplatin in vitro was mediated by caspase‐dependent apoptosis, without any effects from necrosis. In vivo, Nec‐1 was used to inhibit RIP1‐mediated necrotic disease and reduce HL induced by kanamycin and cisplatin.^160^ These results suggest that the harmful factors (ototoxic drugs, ageing and noise exposure) can induce cell death via different PCD pathways. However, the crosstalk between these types of death pathways need to be investigated in future studies.

## 5 CONCLUSIONS

The auditory system is a complex system in which the failure of one component may lead to HL. HL is the most common disease associated with sensory defects, and it affects daily communications, the quality of life of patients and their psychological and mental activities. Although the pathology of HL is very complex, extensive genetic and molecular biological studies have provided considerable insights into the mechanisms underlying hair cell death. PCD involves the traditional death module apoptosis and various other cell death pathways, including autophagy, programmed necrosis, entosis, ferroptosis, lysosome‐dependent cell death and parthanatos. The goal of this review is to provide a general overview of the current knowledge relating to the contribution of PCD in the pathology of hearing impairment, including apoptosis, autophagy and programmed necrosis. This will provide researchers with a summary of the three forms of PCD in HL and allow them to compare and contrast between them. The substantial increase in studies related to PCD, especially those focusing on apoptosis pathways, has contributed to a wealth of knowledge that can facilitate a better understanding of HL pathogenesis and therapeutics. An increased understanding of autophagy and programmed necrosis in recent decades has led to the development of clinical therapies for HL. The occurrence of different types of HL may involve different PCD pathways. Audiological biologists have been trying to understand how these pathways could be mapped and integrated with each other, what global properties are beginning to emerge from interactome network models, and how these properties may relate to HL and its treatment. Therefore, in‐depth studies on the interconnected pathway network comprising the three main functional modules (apoptosis, autophagy and programmed necrosis) are warranted to better understand the pathogenesis and treatment of HL. It is anticipated that with the research conducted on the effect of related target genes, related gene therapy will become the current research hotspot. All these provide new insights to improve global HL.

## CONFLICT OF INTEREST

The authors declare no conflict of interest.

## Data Availability

Data sharing is not applicable to this article as no new data were created or analysed in this study.
